# Efficacy of oseltamivir treatment in influenza virus-infected obese mice

**DOI:** 10.1128/mbio.00887-23

**Published:** 2023-06-21

**Authors:** Rebekah Honce, Jeremy Jones, Victoria A. Meliopoulos, Brandi Livingston, Bridgett Sharp, Leonardo D. Estrada, Lindsey Wang, William Caulfield, Burgess Freeman, Elena Govorkova, Stacey Schultz-Cherry

**Affiliations:** 1 Department of Infectious Diseases, St. Jude Children’s Research Hospital, Memphis, Tennessee, USA; 2 Integrated Program in Biomedical Sciences, University of Tennessee Health Science Center, Memphis, Tennessee, USA; 3 Preclinical Pharmacokinetic Shared Resource, St. Jude Children’s Research Hospital, Memphis, Tennessee, USA; University of Colorado School of Medicine, Aurora, Colorado, USA

**Keywords:** influenza, obesity, antiviral agents, efficacy, viral diversity, interferons

## Abstract

Obesity has been epidemiologically and empirically linked with more severe diseases upon influenza infection. To ameliorate severe disease, treatment with antivirals, such as the neuraminidase inhibitor oseltamivir, is suggested to begin within days of infection especially in high-risk hosts. However, this treatment can be poorly effective and may generate resistance variants within the treated host. Here, we hypothesized that obesity would reduce oseltamivir treatment effectiveness in the genetically obese mouse model. We demonstrated that oseltamivir treatment does not improve viral clearance in obese mice. While no traditional variants associated with oseltamivir resistance emerged, we did note that drug treatment failed to quench the viral population and did lead to phenotypic drug resistance *in vitro*. Together, these studies suggest that the unique pathogenesis and immune responses in obese mice could have implications for pharmaceutical interventions and the within-host dynamics of the influenza virus population.

**IMPORTANCE:**

Influenza virus infections, while typically resolving within days to weeks, can turn critical, especially in high-risk populations. Prompt antiviral administration is crucial to mitigating these severe sequalae, yet concerns remain if antiviral treatment is effective in hosts with obesity. Here, we show that oseltamivir does not improve viral clearance in genetically obese or type I interferon receptor-deficient mice. This suggests a blunted immune response may impair oseltamivir efficacy and render a host more susceptible to severe disease. This study furthers our understanding of oseltamivir treatment dynamics both systemically and in the lungs of obese mice, as well as the consequences of oseltamivir treatment for the within-host emergence of drug-resistant variants.

## INTRODUCTION

Influenza viruses are a seasonal and pandemic threat to human and animal health worldwide (1). Influenza A and B viruses commonly cause seasonal outbreaks in humans, with control measures needed to mitigate its health and economic effects (2, 3). Control of influenza infection is accomplished through vaccination strategies aimed to prevent disease and antiviral courses designed to mitigate symptoms, severity, and spread upon infection (4). Several antivirals have entered clinical use, including adamantanes targeted to the M2 ion channel, neuraminidase inhibitors (NAIs) that block viral release, and endonuclease inhibitors that stall viral replication (5).

However, these antiviral treatment strategies are not always effective. First, resistance to each class of influenza antivirals has emerged, with the adamantanes no longer clinically effective due to widespread resistance (6, 7). While contemporary influenza viruses are largely susceptible to oseltamivir and zanamivir, many NAI-resistant variants have been characterized (7 - 10). Second, poor host responses and delayed treatment with antivirals can impede their efficacy. Host characteristics can promote the emergence of resistant variants, as influenza infection of immunocompromised hosts results in higher rates of antiviral-resistant variants (11 - 16).

The obesity epidemic highlights these dual concerns. Rates of worldwide obesity have nearly tripled in the past 3 decades (17). Obesity results in a chronic state of immunosuppression that impairs the antiviral response to infection, including the type I interferon (IFN) response (18 - 20). Epidemiological data suggest antivirals such as oseltamivir are protective in hosts with obesity (21), yet empirical studies show obese (OB) mice require 10-fold higher doses to achieve complete protection (22, 23). We have previously shown that the obesogenic state impacts viral population dynamics by increasing viral diversity and promoting a more virulent influenza population (19, 24). Thus, we questioned if oseltamivir treatment of OB mice would accelerate viral clearance or lead to the acquisition of antiviral-resistant variants. In these studies, we treated OB mice with the NAI oseltamivir (Tamiflu), and monitored viral clearance over 7 days. Oseltamivir treatment improved viral clearance in wild-type (WT) mice; however, no such improvement was detected in treated, leptin-deficient OB mice. Additionally, we report that this subpar protection promotes the emergence of influenza viral variants with increased neuraminidase (NA) activity and greater oseltamivir resistance. Together, these findings suggest study is warranted on how host characteristics can influence pharmaceutical efficacy (25).

## RESULTS

### Oseltamivir treatment does not reduce viral titers or improve viral clearance in influenza-infected OB mice

**Fig 1:**
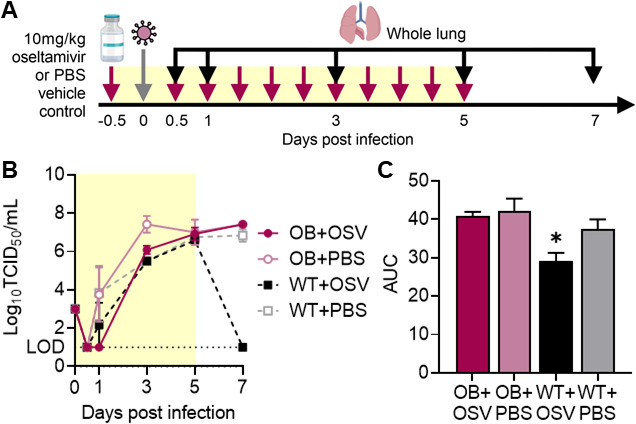
Oseltamivir treatment improves viral clearance in WT, but not OB, mice. (**A**) WT and OB male mice were treated with 10 mg/kg oseltamivir or PBS vehicle control every 12 hours starting 12 hours pre-infection with CA/09 virus. (**B**) Oseltamivir treatment has no impacts on overall viral load but increased viral clearance is observed in WT mice treated with oseltamivir. (**C**) Area under the curve analysis for viral titers in (**B**). Data represented as means ± standard error of the mean (SEM). Data in (**B and C**) represented as means ± SEM and analyzed via (**B**) repeated measures one-way analysis of variance (ANOVA) with Tukey’s multiple comparisons test and in (**C**) with ordinary one-way ANOVA with Tukey’s multiple comparisons test with α = 0.05. Yellow shading indicates active treatment. OSV = oseltamivir phosphate.

We have previously determined that higher doses of oseltamivir are needed to improve survival in OB mouse models (22, 23); however, why the standard dose treatment fails in OB mice is unknown. To answer, we asked if oseltamivir treatment could reduce viral titers or improve viral clearance in WT and OB mice. Beginning 12 hours pre-infection, lean and OB animals were orally gavaged with 10 mg/kg oseltamivir or phosphate-buffered saline (PBS) vehicle control every 12 hours continuing for 5 days post-infection (p.i.) (22). Mice were infected with 1,000 50% tissue-culture infectious dose (TCID_50_) units of A/California/04/2009 (CA/09) H1N1 influenza virus and lungs excised at 0.5, 1, 3, 5, and 7 days p.i. (Fig. 1A). No significant differences in viral titers were detected (Fig. 1B), but area under the curve (AUC) analysis suggests significantly accelerated viral clearance upon oseltamivir treatment for WT mice compared to all other experimental groups (versus WT + PBS *P* = 0.012; versus OB + PBS *P* = 0.0007; versus OB + oseltamivir *P* = 0.0014; Fig. 1C). No trend in accelerated viral clearance was observed for oseltamivir-treated OB mice compared to untreated OB mice in AUC analysis.

### Similar oseltamivir clearance but reduced maximal concentrations in OB compared to lean mice

**Fig 2:**
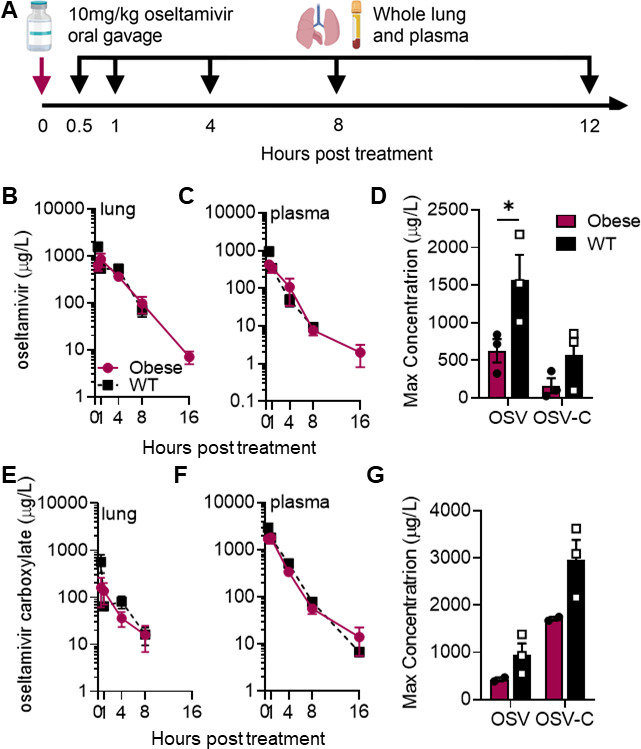
Similar oseltamivir PK parameters in lean and OB mice. (**A**) Naïve WT or OB male mice were dosed once with 10 mg/kg of oseltamivir. Plasma and lungs were collected at indicated time points for bioanalysis of (**B**) oseltamivir and (**C**) oseltamivir carboxylate (OSV-C) in lungs. (**D**) Significantly increased maximum concentration of oseltamivir (*P* = 0.0364) and trends toward increased OSV-C levels were observed in the lungs of WT compared to OB mice. (**E and F**) After 1 dose of oseltamivir, bioanalysis of (**E**) oseltamivir and (**F**) OSV-C in plasma of WT and OB mice. (**D**) Maximum concentration of oseltamivir and OSV-C in plasma of WT and OB mice. Nonsignificant trend toward increased concentration of oseltamivir and OSV-C in WT compared to OB plasma. Data analyzed in (**B, C, E, F**) with ordinary two-way ANOVA and in (**D and G**) with two-way ANOVA and Sidak’s post hoc with α = 0.05. Data represented as means ± SEM.

Appropriate dosage and concentration at the site of infection is crucial for antiviral efficacy; inappropriate levels could lead to severe complications due to treatment failure or the emergence of resistance phenotypes. To determine if the pharmacokinetic (PK) dynamics of oseltamivir and its metabolite oseltamivir carboxylate are altered in the OB mouse, we dosed 10-week-old WT or OB male mice with a single oral gavage of oseltamivir at 10 mg/kg in 100 μL of PBS. Whole lungs and plasma were collected at 0.5, 1, 4, 8, and 16 hours post-treatment and immediately processed for PK analysis (Fig. 2A). There were no practical or significant differences in oseltamivir or oseltamivir carboxylate clearance in plasma and lung between WT and OB mice (Table 1). We observed a similar half-life of oseltamivir (2.12 versus 2.28 hours; Fig. 2B) and its metabolite oseltamivir carboxylate (2.30 versus 3.3 hours; Fig. 2C) in the lungs of OB mice compared to WT mice. Analysis of plasma was similar, with no difference in the half-life of oseltamivir (1.36 versus 1.98 hours, respectively; Fig. 2E) or oseltamivir carboxylate (1.87 versus 2.75 hours, respectively; Fig. 2F). However, the maximum concentration of oseltamivir was significantly increased in both plasma and lung tissue of lean mice compared to OB. In lungs, maximal oseltamivir concentration doubled from an average of 600 μg/L in OB mice lungs to 1,500 μg/L in the lungs of WT mice (*P =* 0.036; Fig. 2D). Plasma showed similar trends (*P* = 0.063; Fig. 2G). Overall, these findings suggest that clearance of oseltamivir is equal between OB and lean mice and most likely has little impact on the observed failure of viral clearance; however, maximal concentrations at the primary site of viral replication could impede overall efficacy.

**TABLE 1:** PK parameters of oseltamivir and oseltamivir carboxylate in plasma and lungs of OB and WT mice

Parameter^*a*^	Unit	Oseltamivir				Oseltamivir carboxylate			
		Obese		Wild-type		Obese		Wild-type	
		Plasma	Lung	Plasma	Lung	Plasma	Lung	Plasma	Lung
C_max_	µg/L	428	839	950	1,570	1,800	162	2,960	565
t_max_	hour	0.5	1	0.5	0.5	1	0.5	0.5	1
AUC_last_	hour·µg/L	1,110	3,290	1,080	3,350	4,810	439	6,150	637
AUC_inf_	hour·µg /L	1,110	3,310	1,100	3,630	4,860	486	6,170	735
Kp_last_	-	0.3374		0.3224		10.96		9.655	
Kp_inf_	-	0.3353		0.3030		10.00		8.395	
K_el_	1/hour	0.35	0.328	0.509	0.304	0.252	0.301	0.372	0.208
t_1/2_	hour	1.98	2.12	1.36	2.28	2.75	2.3	1.87	3.33
CL/F	L/hour/kg	8.99	3.02	9.09	2.75	2.06	20.6	1.62	13.6
V_z_/F	vol/kg	25.7	9.22	17.9	9.05	8.16	68.2	4.36	65.2
C_last_	µg /L	1.99	7.02	9.32	69.1	14.1	15.9	6.8	16.2
t_last_	hour	16	16	8	8	16	8	16	8

^*a*^ Parameters measured in units reported in parenthesis with C_max_ = maximum concentration of analyte; t_max_ = time to reach maximum concentration of analyte; AUC_last_ = area under the concentration–time curve from time 0 to time of last measurement concentration; AUC_inf_ = area under the concentration–time curve from time zero to infinity; Kp_last_ = apparent plasma-to-lung partition coefficient ratio of AUC_last_ in plasma to lungs; Kp_inf_ = apparent plasma-to-lung partition coefficient ratio of AUC_inf_ in plasma to lungs; K_el_ = rate constant; t_1/2_ = elimination half-life; CL/F = time of apparent total clearance of the drug; V_z_/F = apparent volume of distribution during terminal phase; C_last_ = concentration of analyte at last measured time; t_last_ = time at which concentration of analyte was above the lower limit of detection.

### OB-derived viruses are more resistant to oseltamivir treatment *in vitro* compared to those obtained from lean hosts

**Fig 3:**
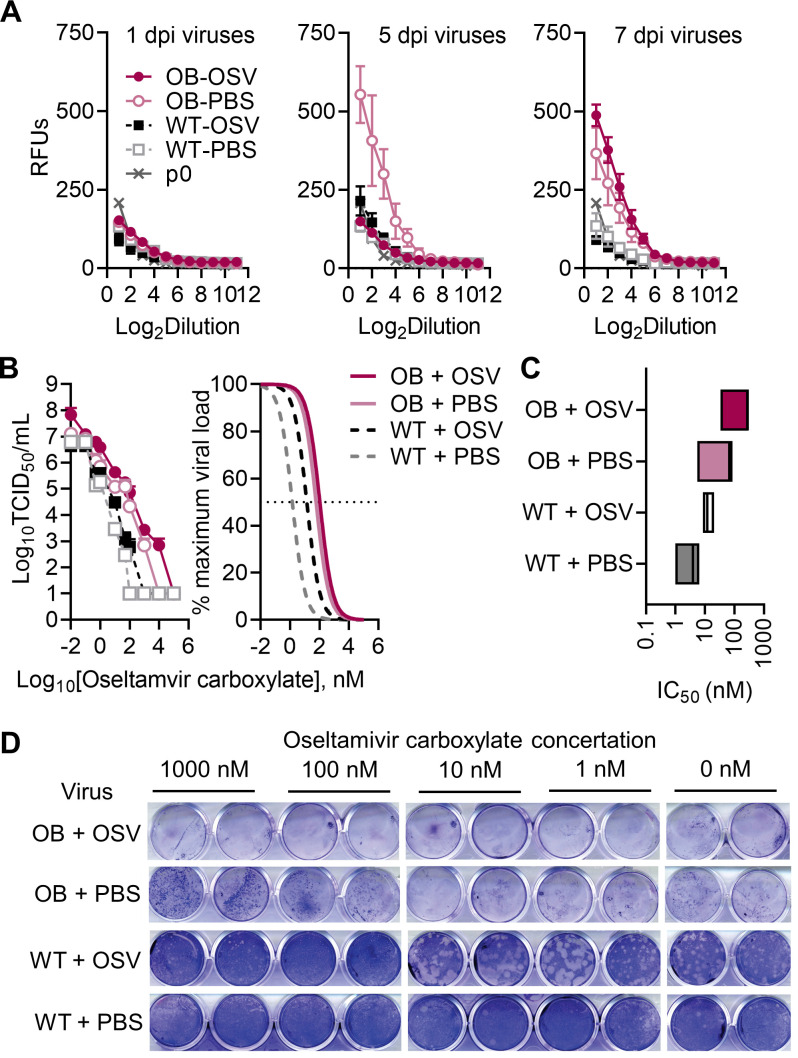
OB-derived viruses are more resistant to oseltamivir carboxylate. (**A**) Relative NA activity is higher in OB-derived viruses. (**B and C**) Indicated viruses were titrated in the presence of increasing concentrations of oseltamivir carboxylate, with (**B**) viral titers determined through TCID_50_ and average nonlinear curve fits of viral loads compared to maximum at no treatment, and (**C**) inhibitory concentrations of oseltamivir carboxylate needed to reduce viral load by 50% compared to no treatment. (**D**) Representative crystal violet staining of plaques derived from indicated homogenized mouse lungs. Images show duplicate wells from a single representative sample assayed across multiple plates in the presence of indicated oseltamivir carboxylate concentrations. Data presented as (**A and B**) means ± SEM and analyzed in (**B**) through area under the curve analysis via one-way ANOVA with Tukey’s multiple comparisons test and nonlinear fits with a nonlinear fit curve to determine half-maximal inhibitory concentration (IC_50_) values reported in (**C**). OSV = oseltamivir phosphate.

While emergence of antiviral resistance is relatively rare, we hypothesized that this delayed viral clearance and the reduced maximal concentrations in the lung tissue could generate resistance to the drug in immunocompromised OB hosts (16, 26). To test this, we compared the NA activity of lean and OB-derived viruses at different days p.i. by MUNANA (27). WT-derived viruses have low NA activity regardless of days p.i. in the presence of oseltamivir. In contrast, the virus isolated from OB host has high NA activity by day 5 p.i. that remains high throughout the course of infection. While the presence of oseltamivir initially suppresses NA activity in OB mice, without impacting overall viral titers (Fig. 1B), by day 7 p.i., viruses emerged with greater NA activity in the presence of the NAI oseltamivir. Initially, viruses derived from all four experimental groups showed similar levels of NA activity as measured by fluorescence (Fig. 3A). By day 5 p.i., virus derived from untreated OB-derived virus showed greater relative fluorescence than other groups. After the removal of treatment, oseltamivir-treated OB mice displayed a rebound in NA activity (Fig. 3A), suggesting a possible selection of higher-NA activity viral variants within oseltamivir-treated OB mice.

To quantitate the difference in potential oseltamivir resistance between WT- and OB-derived viruses, the viruses were titrated on Madin–Darby canine kidney cells (MDCK) cells in the presence of increasing concentrations of oseltamivir carboxylate. Input amounts of infectious virus varied among samples due to their differences in observed titer (Fig. 1), so all were normalized to maximal growth in the dimethyl sulfoxide (DMSO) control condition for direct comparisons of titer reduction. Viruses derived from treated OB mice trended toward higher titers at all experimental conditions compared to both treated and untreated WT-derived viruses (Fig. 3B). AUC analysis showed significantly reduced viral titers across all experimental conditions in treated WT (*P =* 0.0316) and all untreated (WT + PBS, *P* = 0.0173; OB + PBS *P* = 0.0425) mice compared to treated OB mice (OB + oseltamivir). The reported half-maximal inhibitory concentration (IC_50_) for oseltamivir ranges from 0.8 nM to >35 µM (6, 12). Within our experimental conditions, IC_50_ values for all OB-derived viruses were shifted higher compared to WT-derived viruses, with oseltamivir treatment further shifting resistance in both groups (Fig. 3C). Mean IC_50_ values are as follows: OB + oseltamivir, 206.4 ± 86.3 nM; OB + PBS, 52.8 ± 24.4 nM, WT + oseltamivir, 13.7 ± 3.5 nM, WT + PBS 3.8 ± 1.6 nM, clearly shown by crystal violet staining in treated MDCK monolayers (Fig. 3D). In total, OB-derived viruses show greater phenotypic resistance to oseltamivir carboxylate treatment *in vitro* compared to lean-derived viruses, suggesting a potential accumulation of resistant variants in OB-derived influenza populations compared to the viral population in lean animals.

### OB-derived viruses do not have genetic changes commonly associated with antiviral resistance

The observed increases in oseltamivir carboxylate concentration required to reduce viral growth in OB-derived viruses led us to question if resistant variants had emerged in these viral populations. The influenza virus hemagglutinin (HA) and NA segments are genetically plastic allowing for accumulation of potentially resistant single nucleotide variants (SNVs). The HA mutations G155E and D222G as well as the NA mutation H275Y are implicated in reducing oseltamivir efficacy, with several others also suggested as modulators of resistance (28, 29). To determine if these mutations were present in the OB-derived viruses, next-generation sequencing (NGS) was performed and we quantified the overall number of SNVs, entropy, and if any classically NAI-resistant mutants emerged. No consensus changes associated with oseltamivir resistance were detected.

**Fig 4:**
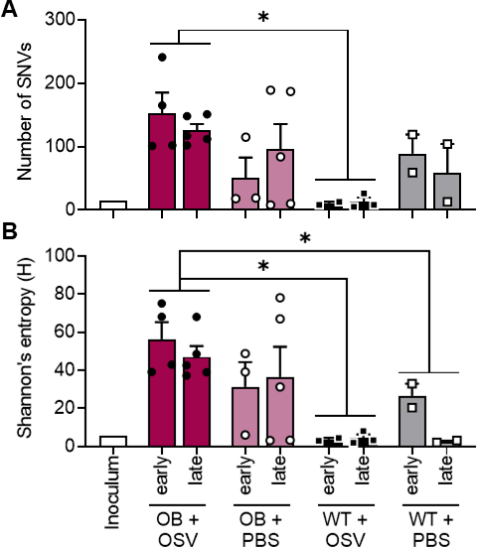
Oseltamivir treatment ablates viral diversity in WT, but not OB, mice. (**A and B**) Viral RNA was extracted from lungs of mice treated with oseltamivir as described in Fig. 2A. Total unique SNVs are significantly higher in oseltamivir-treated OB mice compared to treated WT mice (*P* = 0.0053). (**B**) Oseltamivir-treated OB mice harbor a more diverse viral population compared to oseltamivir-treated (*P* = 0.0035) and PBS control-treated (*P* = 0.0357) WT mice. Statistical comparisons made via two-way ANOVA with Tukey’s multiple comparisons test. Significance was set at α = 0.05. Data represented as means ± SEM. OSV = oseltamivir phosphate.

However, viruses derived from OB mice treated with oseltamivir had significantly increased numbers of unique SNVs compared to viruses derived from WT mice treated with oseltamivir (*P* = 0.0053; Fig. 4A). This translated to increased overall genetic diversity in viruses derived from oseltamivir-treated OB mice. Measures of Shannon’s entropy (H) is reduced in WT treated (*P* = 0.0035) and WT untreated (*P* = 0.0357) compared to oseltamivir-treated OB mice (Fig. 3B). Oseltamivir ablated overall viral diversity in lean mice, with a nonsignificant trend to reduced numbers of SNVs and total Shannon’s entropy in treated lean mice compared to no difference in treated OB mice (Fig. 4A and B).

More SNVs were detected in the HA segment of viruses derived from OB compared to lean treated mice (9 and 4 SNVs, respectively), translating to higher average entropy of 3.27 in OB mice compared to 1.24 in WT mice. Oseltamivir treatment decreased HA entropy in treated WT mice (from 1.98 to 1.24) but not in OB mice, where it increased from 2.30 to 3.27 (Table 1). We detected fewer SNVs in NA. Oseltamivir treatment resulted in one NA variant in OB and three NA variants in lean, compared to two and one in OB and lean PBS control mice, respectively (Table 2). While we noted higher genetic diversity and phenotypic resistance in both oseltamivir-treated and untreated mice, no classical NAI-resistant mutants emerged in the viral populations. However, there is no exhaustive list of mutations that may render oseltamivir treatment less effective, and this would require further study to confirm if we have identified novel resistance markers in this study.

**TABLE 2:** Percentage of detected minor variants in OB and WT mice treated with oseltamivir

Segment	SNV	Mutation	Obese^*a*^		Wild-type^*b*^		Natural prevalence^*c*^
			OSV	PBS	OSV	PBS	
HA	T28C	I10T	6.1				80.8
	T333A	D111E	30.6				>0.1
	A399G	I133M				5.9	0.7
	C432A	D144E		11.0		36.4	>0.1
	A515G	E172G	5.3	8.1	28.1	13.4	78.2
	A517G	N173D		7.1			0.3
	C598T	P200S		5.0			67.6
	T853A	S285T	5.9				>0.1
	T935C	I312T	22.3				>0.1
	A957T	K319N			5.4		>0.1
	G1168A	D390N		7.8			13.7
	T1187C	V396A				8.2	>0.1
	C1193T	S398F				31.8	>0.1
	G1228A	V410I		16.4			>0.1
	T1298A	L433Q			6.0		>0.1
	C1421T	A474V	19.1				>0.1
	A1573G	T525A	10.0				>0.1
	A1579G	I527V			6.7		13.7
	T1610C	V537A	5.8				4.8
	T1654C	F552L	43.6				>0.1
	G1688A	R563K		19.2			0.3
NA	A129T	Q43H	11.6				>0.1
	A149G	N50S				5.0	0.1
	C377T	P126L			7.5		0.1
	T614C	V205A		7.5			>0.1
	G700A	V234I			5.4		0.4
	G1139A	W380		9.7			>0.1
	G1197A	W399			6.7		>0.1

^*a*^ Shannon’s entropy (H) of HA of nontreated versus treated OB mice: 2.30 to 3.27, NA: 0.60 to 0.36.

^*b*^ Shannon’s entropy (H) of HA in nontreated versus treated WT mice: 1.98 vs 1.25, NA: 0.22 to 0.77.

^*c*^ Prevalence of minor variants in human surveillance samples on the Influenza Research Database.

### Role of IFN in oseltamivir-mediated control of influenza virus infection

**Fig 5:**
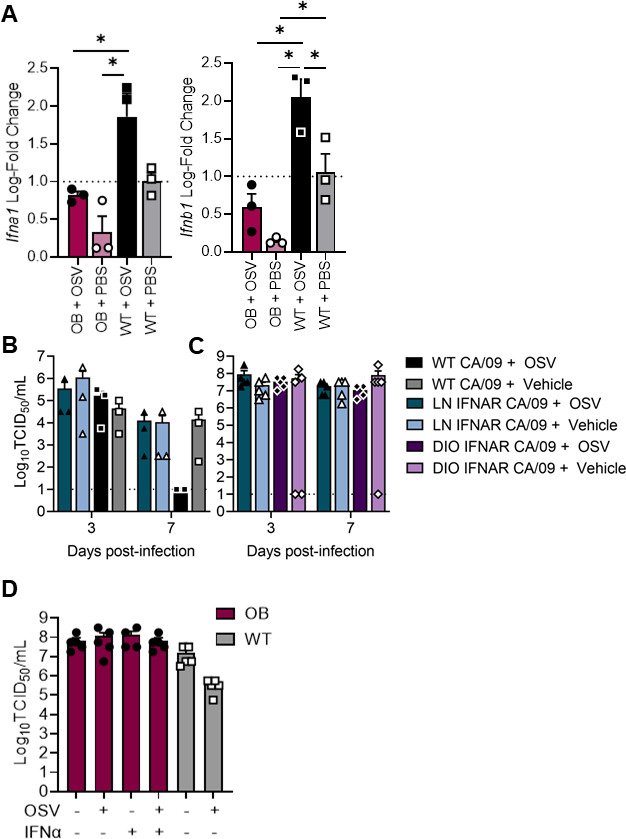
Exogenous IFN treatment fails to improve viral clearance in genetically OB mice. (**A**) IFN expression in lungs of treated or untreated WT and OB mice. (**B and C**) WT and IFNAR^−/−^ mice treated with OSV or vehicle (PBS) and inoculated with CA/09. Lung viral loads quantified at days 3 and 7 p.i. (**D**) WT and OB mice treated with OSV, recombinant IFNα or vehicle (PBS) and inoculated with CA/09. Lung viral loads measured at day 6 p.i. Data represented as means ± SEM and analyzed via ordinary one-way ANOVA with Tukey’s multiple comparisons with α = 0.05. OSV = oseltamivir phosphate.

Obesity is associated with reduced type I IFN responses, and we have previously reported that robust IFN responses restrict influenza genetic diversity (19). We confirmed reduced expression of *Ifna2* and *Ifnb1* in lungs of OB mice compared to WT mice (Fig. 5A), and noted oseltamivir phosphate (OSV) treatment failed to significantly increase levels in OB mice. In contrast, OSV treatment in WT mice further augmented the IFN response (Fig. 5A). To determine if blunted IFN levels alone in the absence of obesity could impact viral clearance, IFNAR^−/−^ or WT male and female mice were treated with 10 mg/kg OSV or vehicle control (PBS) and inoculated intranasally with CA/09 H1N1 virus or PBS as in Fig. 1A. Like OB mice (Fig. 1B and C), OSV treatment showed little effectiveness in reducing viral load in IFNAR^−/−^ mice (Fig. 5B). Only WT mice treated with oseltamivir had reduced viral clearance. IFNAR^−/−^ mice, with and without oseltamivir treatment, had detectable pulmonary viral loads at both days 3 and 7 p.i., phenocopying viral kinetics in OSV-treated genetically OB mice. Diet-induced IFNAR^−/−^ OB mice were no different than IFNAR^−/−^ lean mice (Fig. 5C). Finally, to directly test the hypothesis that a reduced IFN response can decrease the effectiveness of oseltamivir, we treated genetically OB and lean animals with recombinant IFN, which failed to improve pulmonary viral loads at day 6 p.i. (Fig. 5D). Together, our results suggest that IFN is critical for OSV-induced viral clearance in WT mice, but blunted IFN alone does not sufficiently explain the lack of oseltamivir efficacy in genetically OB mice.

## DISCUSSION

In these studies, we demonstrate that oseltamivir treatment reduced viral titers and the emergence of SNVs in WT mice. In contrast, oseltamivir failed to reduce viral titers in OB mice potentially resulting in the emergence of phenotypic resistance to oseltamivir by day 5 p.i. Population-based PK studies have found oseltamivir clearance is accelerated in OB hosts, but not at a biologically significant rate, leading to no weight-based dosage recommendations (23, 28 - 30). However, these studies have relied on plasma concentrations and not the concentration at the respiratory epithelium. No studies to our knowledge have either empirically tested antiviral efficacy in OB hosts or the local concentration of oseltamivir at the site of infection. Here, we find the maximal oseltamivir concentration is reduced at the primary site of influenza virus replication and may impair overall viral clearance in the genetically OB mouse.

While the detailed mechanism of action of oseltamivir is not completely understood, our data in WT mice suggest that complete oseltamivir activity relies on an IFN response. Oseltamivir-treated IFNAR^−/−^ mice, both lean and OB, failed to clear the virus by day 5 p.i. in contrast to the WT mice. This suggests that type I IFN and oseltamivir may also show synergistic benefit in treating seasonal influenza A virus infection, and oseltamivir has been shown to modulate immune responses (31 - 33). These findings led us to hypothesize that the blunted type I IFN response observed in OB mice, in conjunction with reduced maximum concentration of oseltamivir in the pulmonary environment, may result in poor control of viral replication and increase the likelihood for antiviral-resistant variants to emerge, leading to the observed phenotypic resistance (21, 22, 34, 35).

However, exogenous IFN failed to improve viral clearance in oseltamivir-treated OB mice suggesting that oseltamivir-mediated viral clearance may be a complex interaction of PK and immune responses. It is important to note that there remain significant gaps in our understanding of how increased adiposity reduces IFN responses in genetically and diet-induced OB mice and the impact that this might have on exogenous IFN treatment.

We have previously shown that IFN is crucial for controlling the emergence of a diverse viral population that may harbor virulent genotypes (19). The emergence of dominant antiviral-resistant mutations often begin as minor variants in the within-host population, especially in infected, immunocompromised hosts (36, 37). Compounding these risks, oseltamivir-resistant variants in already immunocompromised hosts may complicate an already high-risk medical presentation (38). However, it must also be stated that OB individuals are often met with antifat bias in medical care situations, thus also impacting infectious disease outcomes (21). Weakened immune pressures, including blunted innate and adaptive immunity, coupled with extended shed and potentially higher viral replication may increase the likelihood of adaptive mutations emerging in OB hosts (39). While paradoxically, oseltamivir treatment is not required for the emergence of oseltamivir-resistant variants, adding on this selection pressure in an already compromised situation may prove a perfect storm for viral adaptation. To remedy this, early and appropriate antiviral responses are crucial to control of viral replication, spread, and pathology (19, 21). Treatment delayed as little as 2 days after symptoms onset is ineffective in most hosts, mirroring the sensitive timing for robust action of endogenous IFN responses (4, 40). Further, prolonged treatment may improve results, as stopping oseltamivir treatment at day 5 p.i. led to a rebound in NA activity at day 7 p.i. in viruses derived from treated OB mice (Fig. 3). Identifying the molecular mechanisms behind the blunted antiviral responses due to increased adiposity will be crucial to untangling the multiple impacts obesity may have on individual and public health.

## MATERIALS AND METHODS

### Viruses and titer determination

Eight- to 12-week-old mice were inoculated with 10^3^ TCID_50_ units of CA/09 (H1N1) virus, and viral titer was determined through TCID_50_ assays as previously reported (22).

### Animal husbandry

Eight- to 12-week-old WT C57Bl/6 male (WT) (JAX:000664) and B6.C-Lep*^ob/ob^*/J genetically OB (OB) (JAX:000632) male mice were obtained from Jackson Laboratory. IFNAR knock-out (KO) mice were obtained from Dr. Laura Knoll (University of Wisconsin) and bred inhouse. Knockouts were confirmed by polymerase chain reaction (PCR) using primer sets (IFNAR^−/−^) reported on the Jackson Laboratories website. All animals were housed under standard conditions with food and water provided *ad libitum.* In Fig. 5, IFNAR KO lean (LN) or DIO mice were randomized to the standard control diet (Lab Diets, #5001, 15% fat, 30% protein, 55% carbohydrate) or high-fat diet (Research Diets, #D12492, 60% fat, 20% protein, 20% carbohydrate). All procedures were approved by the St. Jude Children’s Research Hospital Institutional Animal Care and Use Committee and followed the Guide for the Care and Use of Laboratory Animals.

### *In vivo* PK

Plasma and lung tissue PK profiles of prodrug oseltamivir and active metabolite oseltamivir carboxylate was evaluated in male C57BL6 and OB mice (Jackson Labs), approximately 12 weeks in age. OSV was dissolved in PBS at 5 mg/mL for a 10 mL/kg gavage, yielding a 10 mg/kg dose. Terminal blood samples were obtained at various times up to 16 hours post-dose and immediately processed to plasma. A 200 µL aliquot of plasma was then quickly pipetted from each sample and transferred into a separate 2 mL microcentrifuge tube containing 800 µL of ice-cold 15 ng/mL oseltamivir-d3 carboxylate (Toronto Research Chemicals, Lot 14-SBK-47–3, Purity 97%) in methanol, capped, vortexed for 30 seconds and then centrifuged to pellet the precipitated protein. An 800 –950 µL aliquot of the supernatant was then pipetted into an empty pre-labeled microcentrifuge tube, capped, and stored at −80°C until analysis. Following terminal bleeds, animals were perfused with PBS to flush blood from the vasculature. Lungs were then extracted, rinsed with PBS as necessary, and snap frozen in liquid nitrogen. Lungs were then placed in appropriately labeled microcentrifuge tubes in a cooler on dry ice and transferred to −80°C for storage.

### Bioanalysis

Frozen lung samples were weighed in tared 2 mL Lysing Matrix A (MP Biomedical, Santa Ana, CA, USA) tubes and diluted with a 5:1 volume of LCMS grade methanol. The lungs were then homogenized with a FastPrep-24 system (MP Biomedicals, Santa Ana, CA, USA). The homogenization consisted of three 6.0 M/S vibratory cycles of 1 minute each on the FastPrep-24 system. To prevent overheating due to friction, samples were placed on wet ice for 5 minutes between each cycle. The homogenates were then centrifuged at 14,000× g and stored at −80°C until analysis. Plasma and lung homogenate samples were analyzed for oseltamivir (OSV, Cayman Chemical Co., Batch 0458276–29, purity 98%) and oseltamivir carboxylate (Toronto Research Chemicals, Lot 3-SKC-52–1, Purity 98%) with a qualified LC MS/MS assay. Calibration and quality control stock solutions were prepared in DMSO and serially diluted in DMSO to prepare calibration/QC spiking solutions. For the analysis of plasma samples, these spiking solutions were then used to prepare calibration and QC samples in methanol. Methanol calibration and QC samples, 200 µL each, were then pipetted into glass high-performance liquid chromatography (HPLC) vials and evaporated on a CentriVap Console (Labconco) evaporator (40 minutes at 60°C, then 30 minutes at 70°C). The residue was reconstituted in 800 µL of ice-cold 15 ng/mL oseltamivir-d3 carboxylate in methanol as an internal standard. Blank male C57Bl/6 plasma (200 µL) was then pipetted into each vial, immediately vortexed for 1 minute and centrifuged to pellet the protein.

Since the lungs were simultaneously homogenized and protein precipitated in methanol, the calibration and QC samples were prepared by spiking into blank male C57Bl/6 lung homogenate. Aliquots (25 µL) of the standards, QC solutions, and samples were then pipetted into the spiking solution and vortexed to mix. A 2 µL aliquot of the extracted supernatant was injected onto a Shimadzu LC-20ADXR HPLC system via a LEAP CTC PAL autosampler. The LC separation was performed using a Phenomenex Kinetex Polar C18 (2.6 µm, 50 mm × 2.1 mm) column maintained at 50°C with gradient elution at a flow rate of 0.50 mL/minute. The binary mobile phase consisted of water-formic acid (100:0.1 vol/vol) in reservoir A and acetonitrile formic acid (100: 0.1 vol/vol) in reservoir B. The initial mobile phase consisted of 5% B with a linear increase to 55% B in 3 minutes. The column was then rinsed for 2 minutes at 100% B and then equilibrated at the initial conditions for 2 minutes for a total run time of 7 minutes. Under these conditions, oseltamivir carboxylate, IS, and oseltamivir eluted at 1.22, 1.22, and 1.83 minutes, respectively.

Analyte and IS were detected with tandem mass spectrometry using a SCIEX 5500 QTRAP in the positive electrospray ionization (ESI) mode and the following mass transitions were monitored: oseltamivir carboxylate 285.18 to 138.10, oseltamivir-d3 carboxylate 288.20 to 139.20, and oseltamivir 313.20 to 166.20. The method qualification and bioanalytical runs all passed acceptance criteria for non-good laboratory practice (GLP) assay performance. A linear model (1/×2 weighting) fit the calibrators across the 1.00–500 ng/mL range, with a correlation coefficient (R) of ≥0.9973. The lower limit of quantitation, defined as a peak area signal-to-noise ratio of 5 or greater versus a matrix blank with IS, was 1.00 ng/mL. Sample dilution integrity was confirmed. The plasma intra-run precision and accuracy was ≤6.63% confidence variable (CV) and 95.8% to 109%, respectively. The lung homogenate intra-run precision and accuracy was ≤4.48% CV and 94.9% to 111%, respectively.

### PK analysis

Oseltamivir plasma Ct data were grouped by matrix and nominal time point, and the mean Ct values were subjected to noncompartmental analysis using Phoenix WinNonlin 8.1 (Certara USA, Inc., Princeton, NJ, USA). The extravascular model was applied, and AUC values were estimated using the “linear-up log-down” method. The terminal phase was defined as at least three time points at the end of the Ct profile, and the elimination rate constant (K_el_) was estimated using an unweighted log-linear regression of the terminal phase. The terminal elimination half-life (T_1/2_) was estimated as 0.693/K_el_, and the AUC from time 0 to infinity (AUC_inf_) was estimated as the AUC to the last time point (AUC_last_) + C_last_ (predicted)/K_el_. Other parameters estimated included observed maximum concentration (C_max_), time of C_max_ (T_max_), concentration at the last observed time point (C_last_), time of C_last_ (T_last_), apparent clearance (CL/F = Dose/AUC_inf_), and apparent terminal volume of distribution (Vz/F). The apparent plasma-to-lung partition coefficient (Kp_inf_) was estimated as the ratio of the AUC_inf_ in tissue to AUC_inf_ plasma, whereas Kp_last_ was similarly estimated using AUC_last_ values.

### Drug treatment *in vivo*

Oseltamivir was administered by oral gavage at 10 mg/kg free-base equivalencies twice daily for 5 days. At 12 hours after the initial dose, mice were lightly anesthetized with isoflurane and inoculated with 10^3^ TCID_50_ units of CA/09 (H1N1) virus in 25 µL PBS. Control mice received a PBS oral gavage at the same time points. At 0.5, 1, 3, 5, and 7 days p.i., lungs were collected, homogenized in 1 mL PBS, and stored at −80°C for downstream viral titer determination and deep sequencing. For deep sequencing statistical analysis, days 1 and 3 and days 5 and 7 are grouped as early and late points in infection, respectively, due to low copies of viral RNA at very early and very late periods in infection. Recombinant IFN treatment was performed as previously reported (19). Eight-week-old OB and C57BL/6 WT male mice were intranasally infected with 10^3^ TCID_50_ of p0 in 25 µL PBS. Eight hours pre-infection, mice were primed with a 100 µL oral gavage of either 1,000 U recombinant murine IFN-α2 (number 14831262; ThermoFisher) or vehicle control (PBS plus 0.1% BSA). Eight hours post-infection, mice were lightly anesthetized with isoflurane and intranasally treated with 1,000 U recombinant murine IFN-α or vehicle control in 25 µL. Weights and clinical scores were monitored daily for 14 days. Lungs (*n* = three mice per group) were collected at day 3 p.i. and homogenized in PBS with viral titers quantified via TCID_50_. Viral diversity was calculated through deep sequencing and measurements of Shannon’s entropy.

### Drug susceptibility assays

MDCK cells (RRID: CVCL_0422) were maintained in minimum essential medium (MEM; Lonza) supplemented with 2 mM GlutaMAX (Gibco) and 10% fetal bovine sera (Atlanta Biologicals) and grown at 37°C under 5% CO_2_. MDCK cells were seeded in a 12-well or 96-well cell culture-treated plate. Upon confluency, MDCK cells in 96-well plates were inoculated in triplicate with 10-fold serial dilutions of indicated viruses in infection media [MEM, 2 mM GlutaMAX (Gibco), 1% BSA, and 1 µg/mL N-tosyl-L-phenylalanine chloromethyl ketone (TPCK)-treated trypsin] containing increasing concentration of oseltamivir carboxylate (0, 0.1, 0.5, 1, 10, 50, 100, 500, 1,000, and 10,000 nM) or DMSO. Plates were maintained at 37°C under 5% CO_2_ for 3 days, at which time viral titer was determined through hemagglutination of 0.5% turkey red blood cells in PBS. Infectious viral titers were calculated using the Reed–Muench method (33). The dose–response curve was fitted to determine the necessary concentration of oseltamivir to reduce viral titers by 50% (IC_50_). Input amounts of infectious viruses were not equal; instead, we calculated drug susceptibility based on the maximal viral titer in the untreated control condition. Assays were repeated three times with average IC_50_ reported. For modified plaque assays in 12-well plates, MDCK cells were washed twice with PBS, then inoculated at a multiplicity of infection (MOI) = 0.01 with indicated viruses. After a 1-hour adsorption, cells were washed twice with PBS and overlaid with a 1.2% agarose in DMEM mixture containing TPCK trypsin and increasing concentrations of oseltamivir carboxylate as indicated. At day 3 p.i., overlay was removed, and cells were stained with crystal violet to visualized cytopathic effects.

### Quantifying NA activity

The relative NA activity of the oseltamivir-treated and untreated OB- and WT-derived viruses was measured by using the fluorogenic substrate MUNANA (Sigma-Aldrich, St Louis, MO, USA) (27). Twofold dilutions of days 1, 5, and 7 p.i. lung homogenates were prepared in enzyme buffer [32.5 mM of 2-(N-morpholino) ethanesulfonic acid, 4 mM of calcium chloride, pH 6.5] and added in duplicate to a flat-bottom 96-well opaque black plate (Corning). Pre-warmed MUNANA substrate was added to all wells (30 µL/well) to achieve a final concentration of 100 µM. Immediately after adding the MUNANA substrate, the plate was incubated for 1 hour at 37°C. After incubation, the reaction was terminated by addition of 150 μL per well of stop solution (0.014M NaOH in 83% EtOH) and the fluorescence was read at excitation at 355 nm and emission at 460 nm (BioTek). Background-corrected relative fluorescence units were compared to a six-point standard curve of 4-methylumbelliferone (4-MU).

### RNA extraction and qPCR

Total RNA was extracted from whole-lung homogenate using a QIAshredder (number 79654; Qiagen) and RNeasy minikit (number 74104; Qiagen) with DNase treatment. RNA was stored at –80°C prior to the generation of cDNA using SuperScript VILO and qPCR using the QuantiFast SYBR green kit (number 204054; Qiagen) and QuantiTect primer assays for *Ifna2* and *Ifnb1.* Resulting *C_T_* values were normalized to endogenous *Gapdh* expression, and Δ*C_T_* values to average expression of target genes in untreated WT mice with ΔΔ*C_T_* values reported as fold changes over baseline.

### Deep sequencing and bioinformatics

Viral RNA was extracted from 50 µL of whole-lung homogenate or NHBE cell lysates and supernatant on a Kingfisher Flex Magnetic Particle Processor (Thermo Fisher Scientific) by using the Ambion MagMAX-96 AI/ND Viral RNA Isolation kit (Applied Biosystems, cat#AM1834). RNA concentration was measured spectrophotometrically (Nanodrop). Multi-segment RT-PCR (MS RT-PCR) was performed using SuperScript IV One-Step RT-PCR System with Platinum Taq High Fidelity DNA Polymerases (ThermoFisher, cat#12574–035) and influenza-specific universal set of primers (41) (Opti-F1 5´-GTTACGCGCCAGCAAAAGCAGG-3´, Opti-F2 5´-GTTACGCGCCAGCGAAAGCAGG-3´, Opti-R1 5´-GTTACGCGCCAGTAGAAACAAGG-3´). RNA (5 μL) was added and placed into a thermocycler paused at 55°C. The following cycling parameters were followed: 1 cycle of 55°C/2 minutes; 1 cycle of 42°C/60 minutes, 94°C/2 minutes; 5 cycles of 94°C/30 seconds, 44°C/30 seconds, 68°C/3.5 minutes; 26 cycles of 94°C/30 seconds, 57°C/30 seconds, 68°C/3.5 minutes; 1 cycle of 68°C/10 minutes; and then held at 4°C. Five microliters of the reaction was analyzed by 0.8% agarose gel electrophoresis to verify all genomic segments are present, and the reaction purified using the Agencourt AMPure XP Kit (Beckman Coulter) according to manufacturer’s instructions. The concentration of the purified DNA was measured spectrophotometrically prior to storage at −20°C (Nanodrop). DNA amplicons were deep sequenced using Illumina MiSeq technology performed by the St. Jude Children’s Research Hospital Hartwell Center with DNA libraries prepared using Nextera XT DNA-Seq library prep kits (Illumina, cat#FC-131–1024) with 96 dual-index barcodes and sequenced on an Illumina MiSeq personal genome sequencer. SNVs relative to the reference sequence [CA/09 (H1N1)] were determined by mapping reads using the low-variant detection method in CLC Genomics Workbench 12 (41). To determine whether the variants identified have been previously detected in human surveillance samples, we used the protein sequence variance analysis for HA and NA at the Influenza Research Database (42). Comparison of variants was made in reference to CA/09 virus, and relative variant frequencies were calculated by dividing the number of the amino acid variants by the total number of sequences queried.

### Statistical analyses and data visualization

Data were organized in Microsoft Excel and GraphPad Prism 9. Experiment schematics in Fig. 1 and 5 were created using bioRender. Specifics of statistical details for each experiment can be found in the figure legends. All data are displayed as means ± standard error of the mean with stars used to denote statistical significance. Significance was set at α = 0.05.
